# Epidemiology of gestational diabetes mellitus in Latin America and the Caribbean: a scoping review

**DOI:** 10.1080/16549716.2026.2759388

**Published:** 2026-11-10

**Authors:** Adriana Granich-Armenta, Felipe Rangel-Osuna, Alejandra Cantoral, Mónica Ancira Moreno, Christina Anne Vinter, Héctor Lamadrid-Figueroa, Jesper Bo Nielsen, Jens Søndergaard, Fereshteh Baygi

**Affiliations:** a Research Unit of General Practice, Department of Public Health, University of Southern Denmark, Odense, Denmark; b Health Department, Universidad Iberoamericana, Mexico City, Mexico; c Research Unit of Gynecology and Obstetrics, Odense University Hospital, University of Southern Denmark, Odense, Denmark; d Department of Clinical Research, University of Southern Denmark, Odense, Denmark; e Steno Diabetes Center Odense, Odense University Hospital, Odense, Denmark; f Department of Perinatal Health, National Institute of Public Health, Cuernavaca, Mexico

**Keywords:** Gestational diabetes mellitus, prevalence, incidence, risk factors, diagnostic criteria

## Abstract

Gestational diabetes mellitus (GDM) is a major maternal health concern associated with adverse outcomes for mothers and their offspring. In Latin America and the Caribbean (LAC), its burden is heightened by socioeconomic inequalities, limited prenatal care, and high obesity rates, while evidence for other determinants remains fragmented and heterogeneous. This scoping review aimed to identify, describe, and categorize existing research on the prevalence, incidence, determinants, and risk factors of GDM in LAC. We conducted a scoping review following JBI guidance and PRISMA-ScR. Embase, MEDLINE/PubMed, Web of Science, SciELO, and LILACS were searched from inception to December 2025. Eligible studies included observational studies reporting GDM prevalence, incidence, diagnostic criteria, or risk factors in LAC. Two reviewers independently screened records and extracted data. Findings were synthesized descriptively. Fifty‑six studies from nine LAC countries were included, mainly from Brazil and Mexico. Studies were predominantly cross-sectional, cohort, and case-control designs conducted in hospital or prenatal care settings. Diagnostic approaches varied, with IADPSG (39.3%), WHO (25.0%), and ADA (16.1%) as the most frequently used criteria. GDM prevalence ranged from 3.0% to 61.0%, and incidence estimates varied substantially. The most frequently reported determinants and risk factors were advanced maternal age, overweight and obesity, family history of diabetes, previous GDM, prior macrosomia, and hypertension. Behavioral and social determinants were less frequently assessed. Variability in GDM prevalence across LAC highlights the need for standardized regional screening and prospective, population-based research. Reducing the regional GDM burden requires integrating clinical interventions targeting modifiable risk factors and improved continuity of care.

## Paper Context

- **Main findings**: Gestational diabetes mellitus prevalence and incidence varied widely across Latin America and the Caribbean, and advanced maternal age, family history of diabetes, previous gestational diabetes, hypertension, overweight and obesity were consistently identified as risk factors.
- **Added knowledge**: Important evidence gaps remain, including limited data from the Caribbean and Central American countries, few prospective population-based studies, and insufficient assessment of behavioral and social determinants of gestational diabetes.
- **Global health impact for policy and action**: Harmonized screening and diagnostic approaches could strengthen regional surveillance, inform maternal health policies, and help reduce the burden of gestational diabetes and its long-term cardiometabolic consequences.

## Background

Gestational diabetes mellitus (GDM), defined as glucose intolerance first recognized during pregnancy, is one of the most common metabolic complications of pregnancy globally [1]. GDM is associated with adverse maternal and neonatal outcomes including hypertensive disorders [2], cesarean delivery [3], macrosomia [4], and increased risk of future type 2 diabetes [5]. Children of women with GDM are also at increased risk of neonatal complications and long-term metabolic disorders, including obesity and metabolic syndrome [6].

Globally, the prevalence of GDM is estimated to be approximately 14.0% [7], although substantial regional variation exists. According to the International Diabetes Federation, GDM prevalence is estimated to be 10.8% in Africa, 13.9% in Europe and 10.2% in Middle East and North Africa [8]. In North America and the Caribbean, the GDM prevalence reaches 17.9% while in South and Central America it is 12.5% [8]. The highest regional prevalence estimates have been reported in South-East Asia (24.9%) and the Western Pacific region (17.5%) [8].

Several studies from high-income countries have identified advanced maternal age [9], high body mass index (BMI) [10], sedentary behavior [11], and family history of diabetes [12] as key risk factors for GDM [13]. However, it remains unclear whether these associations are consistent across the diverse sociocultural and healthcare contexts found in Latin America and the Caribbean (LAC). Structural determinants such as healthcare access, income inequality, and differences in antenatal care quality may influence both the diagnosis and risk of GDM [14].

In LAC, the burden of GDM is of particular concern given the region’s socioeconomic inequalities [15], limited access to prenatal care [16], {Ye, 2022 #4} and reported prevalences of obesity in pregnant women as high as 40.7% [10], alongside other risk factors such as sedentary lifestyles [17,18]. Despite this, data on the epidemiology of GDM across LAC countries remain limited, fragmented, and heterogeneous. A variety of factors contribute to this inconsistency, including disparities in diagnostic criteria, varying screening approaches, and methodological differences in population-based research [16].

The International Diabetes Federation (IDF) reports highly variable prevalence and incidence estimate of GDM [8], with limited comparability across studies due to differences in diagnostic criteria, screening approaches, and study design [19]. This heterogeneity limits the generation of reliable and comparable estimates of GDM burden across countries [20].

To our knowledge, no previous scoping review has comprehensively mapped the evidence on GDM epidemiology, determinants, and methodological characteristics across the region. Such an overview is needed to identify research gaps, inform future epidemiological studies, and support evidence-based policies and preventive strategies.

This scoping review aimed to map the available evidence and address knowledge gaps by focusing on the following research questions: 1) What is the reported prevalence and incidence of GDM among pregnant women in LAC? 2) What biological, behavioral, and social determinants and risk factors for GDM have been identified in observational studies conducted in the region? 3) What are the methodological characteristics of observational studies on GDM in LAC? 4) What gaps exist in the current literature regarding GDM epidemiology and associated risk factors?

## Methods

### Identification of relevant studies

This scoping review was conducted in accordance with the Joanna Briggs Institute (JBI) methodology [21] and reported following the PRISMA Extension for Scoping Reviews (PRISMA-ScR) guidelines [22]. The protocol was prospectively registered in the Open Science Framework (OSF) Registries (DOI: 10.17605/OSF.IO/KNJZC) on 18 September 2025.

We searched the following five databases from inception to 31 December 2025: Embase, MEDLINE (via Ovid), Web of Science Core Collection (WoS), Scientific Electronic Library Online (SciELO), and Latin American and Caribbean Health Sciences Literature (LILACS). We chose them because they include international and regional literature. Searches were conducted in English, Spanish, and Portuguese using database-specific controlled vocabulary and free-text terms, with normalization of accents and diacritics where applicable.

The search strategy combined terms related to gestational diabetes mellitus and countries within the LAC region. Controlled vocabulary (Mesh, Entree, and DeCS) and free-text terms were used, and search strategies were adapted to each database. No date restrictions were applied. The full search strategy is provided in Supplementary material 1.

### Inclusion and exclusion criteria

#### Inclusion criteria

Studies were included if they met the following criteria: the population comprised pregnant women, including those diagnosed with GDM, and studies were conducted in countries of LAC. The LAC region was defined according to the United Nations regional classification and included countries in Central America, South America, and the Caribbean (Mexico, Guatemala, El Salvador, Honduras, Nicaragua, Costa Rica, Panama, Cuba, Dominican Republic, Colombia, Venezuela, Ecuador, Peru, Bolivia, Brazil, Paraguay, Chile, Argentina, Antigua and Barbuda, Barbados, Belize, Jamaica, Dominica, Grenada, Guyana, Haiti, Saint Kitts and Nevis, Saint Lucia, Saint Vincent and the Grenadines, Suriname, Trinidad and Tobago, Bahamas and Uruguay) [23].

Eligible studies were restricted to observational designs (cohort, case-control, and cross-sectional). Mixed-methods studies were eligible only when their quantitative component used an eligible observational design and reported outcomes relevant to the review. Eligible studies reported GDM diagnosis based on recognized criteria, such as those from the World Health Organization (WHO), American Diabetes Association (ADA), International Association of the Diabetes and Pregnancy Study Groups (IADPSG), Carpenter-Coustan, or national or locally adapted guidelines. Studies had to report outcomes related to GDM, including prevalence, incidence, diagnostic approaches, or associated risk factors in a LAC country or LAC-disaggregated subpopulation.

Studies published up to and including December 2025 were considered. Eligible languages were restricted to Spanish, English, and Portuguese.

#### Exclusion criteria

We excluded randomized controlled trials, intervention studies, and qualitative studies to maintain a strictly observational epidemiological scope. We excluded conference abstracts, case reports, small case series without denominators, editorials, commentaries, narrative reviews, protocols, and clinical trials in which interventions precluded estimation of the underlying occurrence of GDM. Studies exclusively focused on pregestational diabetes (type 1 or type 2) without separate reporting for GDM were excluded. Records with ambiguous GDM definitions were retained only if diagnostic criteria could be identified or reasonably inferred from national or international guidelines cited by the authors. Studies conducted outside LAC or not reporting data relevant to the review questions were excluded.

### Study selection and data extraction

All records were imported into a reference manager EndNote 21.5 [24], duplicates were removed, and records uploaded to the Covidence systematic review software for screening [25]. Study selection was conducted in two stages (title/abstract and full-text screening) by two independent reviewers against the eligibility criteria. When a study was deemed potentially relevant by at least one reviewer, the full text was independently reviewed by two reviewers. Reasons for exclusion at the full-text stage were documented and summarized in a PRISMA-ScR flow diagram. Any discrepancies between reviewers were resolved through discussion, and when consensus could not be reached, a third reviewer was consulted. For studies where the full text was not readily available, efforts were made to obtain the article through institutional access and other sources. Studies for which the full text could not be retrieved were excluded and documented accordingly.

Data extraction was conducted using a standardized form that was piloted prior to the start. Two reviewers independently extracted data, including: authors, year, country, study design, setting, sample size, population characteristics, diagnostic criteria, screening approach, reported prevalence or incidence, determinants/risk factors assessed, effect estimates (where available), and key methodological limitations. Diagnostic criteria were extracted as reported by the original studies and categorized according to the underlying diagnostic framework (IADPSG, WHO, ADA, Carpenter-Coustan, ALAD, or national guidelines). When available, information on screening strategy (universal versus risk-based screening) and diagnostic approach (one-step versus two-step testing) were also extracted because these factors may substantially influence reported prevalence estimates and comparability across studies [26–28].

### Quality appraisal and synthesis

Consistent with established scoping review methodology, no formal risk-of-bias assessment was conducted [22]. Data were synthesized using a descriptive and narrative approach. Studies were organized by country within LAC and further stratified by diagnostic criteria and study design (cross-sectional, cohort, and case-control). Prevalence and incidence estimates were summarized descriptively as reported in the included studies, without statistical pooling.

To address heterogeneity, findings were compared across studies based on differences in diagnostic criteria, screening strategies, and study settings to facilitate interpretation of variations in prevalence and incidence estimates. Reported determinants and risk factors were grouped into predefined domains: demographic, anthropometric, obstetric, behavioral, clinical, and social. Obstetric factors included pregnancy-related characteristics and reproductive history, such as parity, previous GDM, prior macrosomia, and previous cesarean delivery. Clinical factors included non-obstetric medical and metabolic conditions, biomarkers, and comorbidities, such as hypertension, dyslipidemia, insulin resistance, elevated fasting glucose, and inflammatory markers.

## Results

### Study selection

A total of 4,384 records were identified through database searching. After removing duplicates and ineligible records before screening, 991 unique records remained for title and abstract screening. Following screening, 758 articles were excluded based on irrelevance to gestational diabetes mellitus prevalence, incidence, or risk factors in LAC. A total of 233 full-text articles were assessed for eligibility. Of these, 177 studies were excluded for the following reasons: duplicates (*n* = 8), irrelevant outcomes (*n* = 36), non-original research (*n* = 57), population not meeting the inclusion criteria (*n* = 20), postpartum recommendations (*n* = 1), post-GDM consequences (*n* = 12), incomplete full-text availability (*n* = 41), and absence of data on GDM prevalence, incidence, screening, or determinants (*n* = 2). Finally, 56 studies met the inclusion criteria and were included in this scoping review [29–84] (Figure 1).

**Figure 1.**
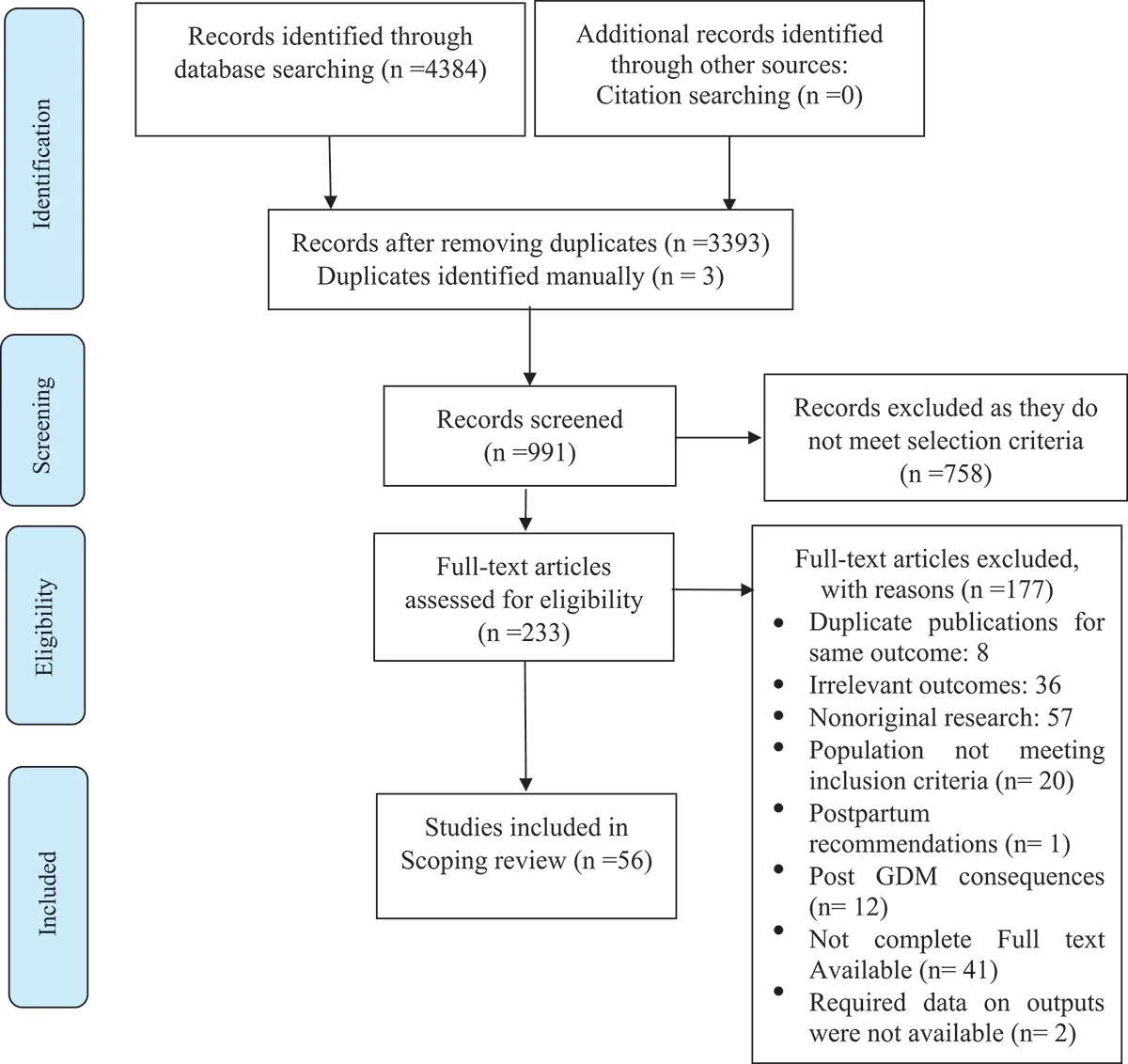
PRISMA diagram for searching resources.

### Characteristics of included studies

Table 1 summarizes the characteristics of the 56 included studies.

**Table 1.** Characteristics of studies included in the scoping review of GDM in Latin America and the Caribbean.

Author (Year)	Country	Study design	Study setting	Data source	Study period	Sample size	Population Characteristics	Maternal Age Mean/Range	Screening criteria	Reported prevalence	Reported incidence
Mella, 1990 [29]	Chile	Cross-sectional	Primary maternal-child health care centers, Santiago	Questionnaires & medical records	1982–1984	680	Pregnant women attending routine prenatal care	29.5	Other (pre-WHO pre-IADPSG criteria)	4.3%	
Agudelo, 1996 [30]	Colombia	Cross-sectional	High-risk obstetric clinic, Medellín	Medical Records	1980–1992	166	High-risk pregnant women, tertiary referral patients	NR	WHO	48.8%	
Forsbach, 1997 [31]	Mexico	Cross-sectional	Antenatal clinic and delivery unit, Monterrey	Medical Records	NR	667	Pregnant women attending an IMSS clinic/unit	18–44	WHO	3.0%	
Reichelt, 1998 [32]	Brazil	Cohort Study	General prenatal care units in six state capitals	Questionnaire, anthropometric measurements, and OGTT	1991–1995	5579	General prenatal population, aged 20 years and older, gestational age 24–28 weeks	27.9	WHO	7.6%	
Corrêa, 2004 [34]	Brazil	Cohort Study	University Hospital Pedro Ernesto, Rio de Janeiro	Medical Records	2001–2002	50	Prenatal Care population attending the University hospital	29.6	WHO	42%	
Belmar, 2004 [33]	Chile	Cohort Study	Hospital Clínico Universty Católica, Santiago	Medical Records	2002–2003	7329	Universal pregnant screening population in university hospital	32.1	WHO and ADA		3.2% (WHO) 7.72% (ADA)
Buteler Maria, 2013 [35]	Argentina	Cross-sectional	Hospital Materno Neonatal, Córdoba	Medical Records	2009–2011	165	Patients in the Córdoba Diabetes Program	NR	WHO	61.0%	
Rodríguez-Fernández, 2014 [40]	Cuba	Cross-sectional	Hospital Docente Ginecobstétrico, Camagüey	Medical Records	2009–2012	929	Pregnant women diagnosed with diabetes pregestational and GDM.	34	WHO	NR	NR
Alves, 2014 [36]	Brazil	Cross-sectional	Public hospitals in Petrolina and Juazeiro	Medical Records	2011–2012	1340	Public health services prenatal users	24.5	Brazilian Ministry of Health	14.7%	
Flores-Padilla, 2014 [37]	Mexico	Case-Control	Family Medicine Units (IMSS), Ciudad Juárez	Medical Records	January 2011–December 2011	232	Pregnant women attending primary care at IMSS.	28.6	ADA	3.9%	
Monroy, 2014 [39]	Mexico	Case-Control	Women’s Specialty Clinic, SEDENA, Mexico City	Questionnaires, obstetric and genetic data	NR	213	Patients at a military specialty clinic.	30	ADA	NR	NR
Guimaraes, 2014 [38]	Brazil	Cross-sectional	Center for Fetal Medicine (UFMG), Belo Horizonte	Medical Records	July 2008- August 2009	80	Pregnant Women and those with GDM	31.7	IADPSG	NR	NR
de Melo, 2015 [42]	Brazil	Case-Control	Public health institutions in Curitiba, Paraná	Medical Records	NR	400	Pregnant women patients attending public health institutions in Curitiba	32	IADPSG	14.0%	NR
Cruz, 2015 [41]	Cuba	Case-Control	América Arias University Maternity Hospital, Havana	Medical Records	October 2002-December 2012	236	Women with history of macrosomic infants.	NR	WHO/National guidelines	NR	NR
Barbieiri, 2016 [44]	Brazil	Cross-sectional	Public Health System of Ribeirao Preto	Questionnaires, anthropometric, dietary recalls, and medical records	March 2011–November 2012	799	Women receiving prenatal care within the Brazilian Unified Health System.	28	WHO	19.0%	NR
Reginatto 2016 [46]	Brazil	Cross-sectional	Public hospital in Santa Catarina	Medical Records and direct placental measurement	May 2012–February 2013	20	Mothers at delivery in a public hospital	30.5	WHO	NR	NR
Nascimento 2016 [45]	Brazil	Cohort	Prenatal clinic at the Instituto IMIP, Recife	Questionnaires, medical records and anthropometric measurements	November 2011–February 2014	841	Low-Income prenatal patients	26.2	IADPSG	10.8%	NR
Gascho, 2017 [47]	Brazil	Cross-sectional	Maternidade Hospital Darcy Vargas, Joinville	Medical Records	NR	392	Public maternity patients, including high-risk outpatient clinic users.	31.1	ADA	NR	NR
Guerra, 2018 [49]	Brazil	Cross-sectional	University Hospital maternity unit, Niterói	Medical Records	January 2013–December 2013	178	Pregnant women in a university maternity setting.	27.1	Brazilian Ministry of Health	14.0%	NR
Ruiz-Hoyos, 2018 [50]	Colombia	Cohort	Level I public healthcare center, Armenia	Biochemical markers	February 2015–July 2016	370	Primary care prenatal patients	22.9	IADPSG	4.7%	NR
Aldrighi, 2018 [48]	Brazil	Cross-sectional	University Hospital in Curitiba, Paraná	Medical Records	September 2015–January 2016	223	Women aged 35–45 receiving prenatal care	35–45	Brazilian Ministry of Health	21.5%	NR
Larrabure-Torrealva, 2015 [43]	Peru	Cross-sectional	National Maternal Perinatal Institute (INMP), Lima	Structured interviews, medical records, OGTT	February 2013–June 2014	1300	Patients at a national referral center for maternal health.	28.9	IADPSG	15.8%	NR
Morais, 2019 [58]	Brazil	Cross-sectional	Basic Health Unit, Lajeado	Structured questionnaires	April–May 2017	20	Women receiving prenatal care	15–35 and 35 and older	Brazilian Ministry of Health	5.0%	NR
Barros, 2019 [51]	Brazil	Case Control	Maternity School of the Federal University of Rio de Janeiro	Medical Records	2009–2015	416	Pregnant women in a university teaching maternity.	NR	IADPSG	48.1%	NR
Hernández, 2019 [56]	Honduras	Cross-sectional	Health Center of San Antonio de Oriente	Medical Records	2015–2016	98	Semi-rural prenatal population.	25.7	ADA-WHO	4.0%	NR
Siqueira, 2019 [60]	Brazil	Case Control	Dept. of Obstetrics, UNIFESP, São Paulo	Medical Records	NR	157	GDM pregnant women and healthy controls attending University Hospital	33	Brazilian Ministry of Health	NR	NR
TuescaMolina, 2019 [61]	Colombia	Cross-sectional	Public health centers, Barranquilla	Medical Records	January 2012–December 2014	1880	Pregnant women who attend the Prenatal care visits.	NR	IADPSG	8.7%	
Chávez-García, 2019 [52]	Mexico	Cross-sectional	Hospital General Regional No. 1, IMSS, Ciudad Obregón	Questionnaires and medical records	November 2017–January 2018	93	Pregnant women attending the Second-level care at IMSS system.	27.8	IADPSG	NR	NR
Pent, 2019 [59]	Argentina	Cross-sectional	Endocrinology outpatient clinic, Hospital Centenario, Rosario	Biochemical markers and medical records	August 2017–July 2018	44	Outpatient pregnant women at an endocrinology specialty clinic	31	ALAD	NR	NR
Villota-Burbano, 2019 [62]	Mexico	Cross-sectional	Hospital de la Mujer, Mexico City	Medical Records	March 2014–December 2017	459	Women attending a high-risk maternal clinic in Mexico City	31.1	IADPSG	NR	NR
Freitas, 2019 [55]	Brazil	Case-Control	Hospital Nossa Senhora da Conceição, Tubarão	Questionnaires and medical records	May 2018–August 2018	140	Hospital-based prenatal population in Southern Brazil	32.8	Brazilian Ministry of Health	NR	NR
Martinez-Cruz, 2019 [57]	Mexico	Cohort	National Institute of Perinatology -INPer, Mexico City	Medical Records	January 2010–December, 2014	282	Tertiary care patients at a national referral institute	29.9	IADPSG	NR	NR
Correa, 2019 [53]	Chile	Case Control	Obstetrics and Gynecology Unit, Clínica Dávila, Santiago	Medical Records, Biochemical markers	NR	96	Pregnant women attending the private health setting in Santiago	32.6	IADPSG	NR	NR
Dantas, 2019 [54]	Brazil	Cross-sectional	Hospital Maternidade Darcy Vargas, Joinville	Medical Records, Biochemical markers	September 2015–January 2017	238	Singleton pregnancies Age > 18 years	32.2	ADA	NR	NR
Reyes-Muñoz, 2020 [65]	Mexico	Cohort	Private and public practices; INPer, Mexico City	Medical Records	2010–2019	144	Women with ≥3 risk factors for GDM	31	IADPSG		29.2% (Group 1Supplemented)47.9% (Group 2 control)
Preciado 2020 [64],	Colombia	Cohort	Clínica Universitaria Bolivariana, Medellín	Medical Records	January 2012–December 2015	197	Pregnant women with GDM	31	IADPSG	NR	NR
DosSantos, 2020 [63]	Brazil	Cross-sectional	Basic health care units, Caxias do Sul	Medical Records	January–December 2016	2313	Primary care users across the municipal health network.		IADPSG	5.4%	NR
Olivares, 2021 [68]	Argentina	Ambispective observational	Interzonal Public Hospital ‘Dr. Lucio Molas’, Santa Rosa	Medical Records	2013–2019	44	Pregnant women in a public interzonal hospital.	28–30	IADPSG	3.7%	NR
Argerich 2021 [67]	Argentina	Cross-sectional	Hospital Perrupato, Mendoza	Medical Records	2015–2019	296	Pregnant women aged > 18 years	28–32	ALAD	NR	NR
Almeida 2021 [66]	Brazil	Cohort	Hospital das Clínicas, Univ. of São Paulo	Questionnaires, medical records	May 2017–October 2018	172	Pregnant women aged ≥ 18 years, Singleton pregnancies, Diagnosed with GDM	NR	WHO	NR	NR
Pagotto, 2022 [69]	Argentina	Cohort	Hospital Italiano de Buenos Aires	Medical Records	June 2015–June 2018	7711	Pregnant women aged ≥ 18 years, Singleton pregnancy	34.4	ALAD	NR	7.6%
Pavesi, 2023 [71]	Brazil	Cross-sectional	Public maternity hospitals, Santa Catarina	Questionnaires, medical records	January–December 2019	3580	Lived in Santa Catarina during pregnancy, prenatal care and delivered in the SUS, singleton births ≥ 22 weeks gestation	20–34	Brazilian Ministry of Health	9.4%	NR
Rovira 2023 [72]	Argentina	Cohort	Hospital Británico de Buenos Aires	Medical Records	January 2018–September 2020	260	women over 18 years of age with GDM	34.5	ALAD	NR	NR
Zavalza-Gomez, 2023 [73]	Mexico	Cross-sectional	Tertiary care hospital in Guadalajara, Mexico	Medical Records	January–December 2018	3510	Pregnant Women undergoing OGTT between 24–28 weeks.	30	WHO and IADPSG	NR	32.7%
Juvinao-Quintero, 2023 [70]	Peru	Cross-sectional	Instituto Nacional Materno Perinatal, Lima, Peru.	Medical Records, Biochemical markers	February 2013–June 2014	527	Women ≥ 18 years, those who initiated prenatal care before 28 weeks of gestation	29.5	IADPSG	23.0%	NR
Garcia-Alvarado 2024 [74]	Mexico	Cross-sectional	Hospital Materno Perinatal ‘Mónica Pretellini Sáenz’, Toluca, State of Mexico, Mexico.	Medical Records, dietary assessment	January 2018–September-2020	42	Pregnant women aged 18–41 years attending outpatient medical and nutritional consultation.	28.6	ADA	NR	NR
Gurza 2025 [75]	Mexico	Cohort	Instituto Nacional de Perinatología INPer, Mexico City, Mexico	Medical Records	2017–2019	333	Pregnant women receiving prenatal care at INPer first trimester, 18 years old and older.	30.8	IADPSG	22.9%	NR
Perichart-Perera 2025 [76]	Mexico	Case-Control	National Institute of Perinatology, Mexico City, Mexico	Medical Records, Biochemical markers	2017–2023	108	Pregnant women enrolled during the first trimester.	31.1	IADPSG	NR	NR
Febres Balestrini, et al (2000) [77]	Venezuela	Cohort	Maternidad ‘Concepción Palacios’ and prenatal centers of the Venezuelan Social Security Institute in Caracas, Venezuela	Prenatal clinic participants; glucose screening and oral glucose tolerance testing; follow-up after pregnancy	1992–2000	3,070	Pregnant women attending prenatal care clinics in Caracas	28	WHO	NR	NR
Nava (2011) [84]	México	Case control	Hospital Materno Perinatal ‘Mónica Pretelini’, Toluca, Mexico	Hospital-based pregnant women undergoing OGTT screening	January 2009 – March 2010	489 pregnant women	Pregnant women aged 13–44 years attending public hospital care in Toluca, Mexico	13–44	ADA	13.0%	NR
Camarena-Pulido (2018) [78]	Mexico	Cross sectional	Hospital Civil de Guadalajara, Mexico	Clinical patients and blood samples	January 2014 – January 2017	784	Pregnant woman with and without GDM	GDM: 30 ± 3.3 non-GDM: 26 ± 5.2	Other	NR	NR
Nicolosi (2020) [79]	Brazil	Cohort	Brazilian public tertiary obstetrical referral center (Botucatu Medical School/UNESP, Brazil)	Prospective cohort database from Perinatal Diabetes Research Center	2008–2014	569	Singleton pregnant women receiving care at a Brazilian referral center	Not clearly reported overall. Risk category analyzed: ≥25 years	IADPSG	36.4%	NR
Collantes-Gutierrez (2020) [80]	Mexico	Case control	Hospital Universitario de Puebla, outpatient prenatal clinic, Gynecology service			50	25 women with GDM and 25 controls without GDM, matched by age and BMI	Without GDM: 30.92 ± 5.06 years With GDM: 32.32 ± 5.52 years	ADA	NR	NR
Pagotto (2020) [81]	Argentina	Cross sectional	Hospital Italiano, Buenos Aires, Argentina		January, 01st 2007–December 31st, 2018	33,985	Pregnant women > 18 years attended at Hospital Italiano, of Buenos Aires		Other	8.5%	NR
Carvalho (2022) [82]	Brazil	Cohort	Federal University of São Paulo (Unifesp), Brazil; gestational diabetes and normal gestation outpatient clinics	Clinic patients; questionnaires; anthropometric evaluation; laboratory blood samples; OGTT	December 2018–August 2020	132 pregnant women	Pregnant women aged > 18 years with BMI ≥ 25 kg/m^2^; women with and without GDM	Without GDM: 28 ± 6 years With GDM: 32 ± 6 years	IADPSG	NR	NR
Zulueta, (2023) [83]	Mexico	Cohort	Six primary healthcare facilities in Hidalgo, Guanajuato, and Mexico City (CME cohort) Mónica Pretelini Sáenz Maternal Perinatal Hospital, Toluca, Mexico (validation cohort)	Prospective cohort data, OGTT measurements, clinical questionnaires, and genomic DNA samples	8 May 2019–18 May 2021	CME cohort: 576 women 107 GDM cases 469 controls Validation cohort: 32 GDM cases 199 controls	Pregnant Mexican women without pregestational T2D and <28 weeks gestation	CME cohort: Cases: 28.64 ± 6.56 years Controls: 26.06 ± 5.98 years Cases: 29.13 ± 6.61 years Controls: 25.60 ± 6.72 years	IADPSG	CME cohort: 107/576 = 18.6% Validation cohort: 32/231 = 13.9%	NR

ALAD: Latin American Diabetes Association, NR: Not Reported, information not provided by the study, OGTT: Oral Glucose Tolerance Test, IADPSG: International Association of Diabetes and Pregnancy Study Group, WHO: World Health Organization.

The number of publications increased progressively after 2010, with the majority published between 2015 and 2025, reflecting growing research interest in GDM in LAC. Brazil contributed the largest number of studies (*n* = 21), followed by Mexico (*n* = 15), Argentina (*n* = 7), Chile (*n* = 3), Colombia (*n* = 4), Peru (*n* = 2), Cuba (*n* = 2), Honduras (*n* = 1), and Venezuela (*n* = 1). Most studies were published in English, particularly those conducted in Brazil (*n* = 15) and Mexico (*n* = 7) (Figure 2).

**Figure 2.**
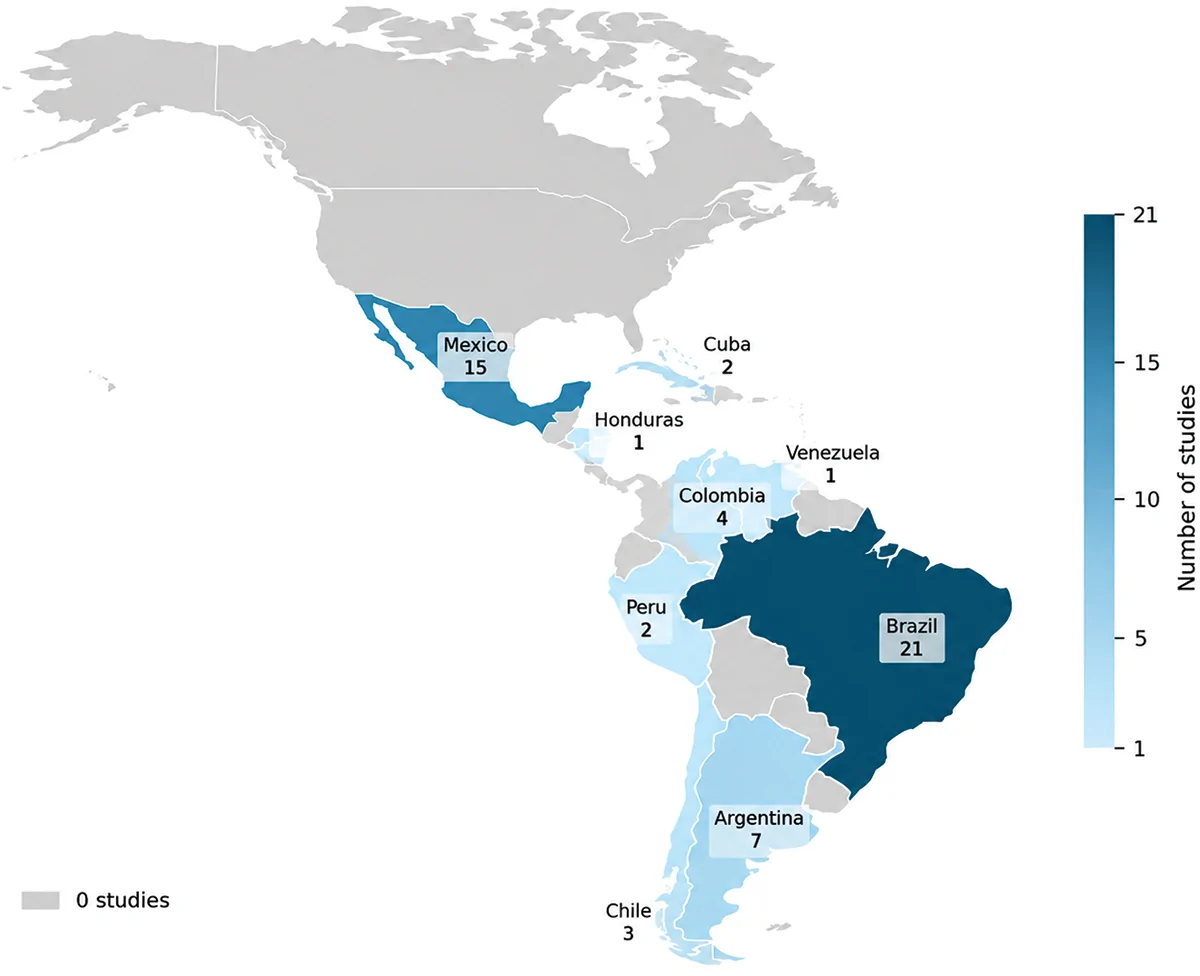
Geographic distribution of the included studies on GDM in Latin America and the Caribbean.

Most studies were conducted in hospital-based prenatal care settings, including tertiary referral hospitals, public health systems, high-risk pregnancy clinics, and primary care centers [29–76,84]. Data from the included studies came from medical records and health databases [29–31,33–49,51–53,55,56,59–64,66–76], interviews and questionnaires [29,32,36,39,43–45,52,55,58,66,70,71,82,83], and clinical laboratory assessments and anthropometric measurements [32,34,37,39,43–45,50,53,54,59,70,73,76,78,82,83]. All included studies were observational in design, cross-sectional studies were the most common (*n* = 28) [29–31,35,36,38,40,43,44,46–49,52,54,56,58,59,61–63,67,70,71,73,74,78,81] followed by cohort studies (*n* = 16) [32–34,45,50,57,64–66,69,72,75,77,79,82,83], case-control studies (*n* = 11) [37,39,41,42,51,53,55,60,76,80,84], and one study used a retrospective-prospective cohort [68]. The sample sizes of the included studies varied by design, ranging from 20 to 33,985 participants in cross-sectional studies, 50 to 7,329 in cohort studies, and 50 to 489 in case-control designs. Most studies included pregnant women aged 18 years or older.

Regarding the diagnostic criteria for GDM, IADPSG was the most frequently utilized standard, reported in 22 studies [38,42,43,45,50–53,57,61–65,68,70,73,75,76,79,82,83], followed by the WHO criteria in 14 studies [30–35,40,41,44,46,56,66,73,77]. Some studies applied more than one diagnostic framework, including combinations of WHO and ADA criteria [33,56] and WHO and IADPSG criteria [73]. Accordingly, these studies are represented in more than one diagnostic-criteria category. Among these, some studies applied the WHO-2013 recommendations, which largely adopted the IADPSG glucose thresholds for the diagnosis of GDM. Other clinical standards identified included the ADA criteria [33,37,39,47,54,56,74,80,84], O’Sullivan and Mahan thresholds [29,37], and regional guidelines such as those from the Brazilian Ministry of Health [36,48,49,55,58,60,71] and the Latin American Diabetes Association (ALAD) [59,67,69,72].

### Magnitude and distribution

Table 1 summarizes the prevalence of GDM reported in the included studies. Overall, reported prevalence varied widely across countries and study settings, ranging from 3.0% to 61%, within and between countries. Among studies using IADPSG criteria, prevalence ranged from 3.7% to 48.1%, whereas studies using WHO criteria reported prevalence ranging from 3.0% to 61.0%. These wide and overlapping ranges show substantial variation in prevalence estimates across studies, even within the same diagnostic criteria. Lower prevalence estimates were reported in several studies conducted in Mexico, Argentina, Honduras, Chile and Colombia, including 3.0% in Mexico (*n* = 667) [31], 3.7% in Argentina (*n* = 44) [68], and 4% in Honduras (*n* = 98) [56], 4.3% in Chile (*n* = 680) [29] and 4.7% in Colombia (*n* = 370) [50]. Similar low prevalence estimates were observed across studies conducted in urban hospital settings in the countries, except Honduras, although the study years differed. Differences in prevalence estimates may reflect variation in study populations, healthcare settings, and diagnostic criteria.

Moderate prevalence estimates were observed in multiple studies conducted across Brazil, Peru, and Mexico, with values ranging between approximately 9% and 23%. For example, prevalence estimates of 10.8% were reported in both Mexico and Brazil [45], while other studies in Brazil reported prevalence values of 14.0% [49], 14.7% [36], 19% [44] and 21.5% [48]. Studies conducted in Peru reported prevalence values of 15.8% [43] and 23% [70]. Studies conducted in Mexico reported intermediate prevalence values including 13.0%, 18.6%, 22.9% and 22.9% in different subpopulations and healthcare settings [75]. Higher prevalence estimates were reported mainly in studies involving high-risk populations, tertiary referral centers, or specialized diabetes clinics. These included prevalence values of 42.0% in Brazil [34] and 48.1% in Brazil [51], 48.8% in Colombia [30], and 61.0% in Argentina [35]. In Mexico, prevalence estimates ranged from a very low values (3.0%) to moderate levels in other studies, reflecting differences across populations, study populations, healthcare settings, diagnostic criteria, and screening approaches. Biological factors such as being overweight and obesity which are well-established risk factors for GDM, may also contribute to this variability [37,84].

#### Incidence of GDM

Table 1. The reported incidence of GDM varied substantially across the included studies according to study design, population characteristics and diagnostic criteria. In Chile, one study reported an incidence of 3.2% using WHO criteria and 7.7% using ADA criteria [33].

Population-based and hospital-based cohort studies also showed considerable variability. In Argentina, a large cohort study reported an incidence of 7.6%, whereas a Mexican tertiary-care study reported one of the highest incidences estimate at 32.7% [69,73]. In another Mexican study reported GDM incidences of 29.2% in the supplemented group and 47.9% in the control group [65], the latter representing the highest incidence reported among the included studies.

### Determinants and risk factors of GDM

The biological, behavioral, and social determinants of GDM identified in the included studies are summarized in Table 2. Across the region, biological risk factors were the most consistently reported, being addressed in approximately 93% of the included studies, particularly advanced maternal age [29,32,33,39,40,42,43,45,48,51,55,61–63,67,69,74,77–84], overweight and obesity [31,35,37,41,43,45,47,49,55,57,59,61–63,66,68–70,81,82,84], elevated body mass index (BMI) [36,37,39,42,44,50,54,60,63,64,66,67,72–76,78–80,83], and family history of diabetes [31,35,37,42–45,55–57,61,62,66,68,70,72–74,79,80,83,84]. Maternal overweight and obesity, reflected in elevated pregestational BMI and excessive gestational weight gain consistently emerged as strong predictors of GDM occurrence.

**Table 2.** Summary of reported risk factors and determinants of GDM in Latin America and the Caribbean.

Author (Year)	Country	Biological Risk Factors	Behavioral Risk Factors	Social Determinants
Mella, 1990 [29]	Chile	Advanced maternal age; elevated fasting glucose	–	–
Agudelo, 1996 [30]	Colombia	Maternal hypertension; gestational diabetes, Non-insulin-dependent diabetes	–	–
Reichelt, 1998 [32]	Brazil	Maternal age; parity.	–	–
Febres Balestrini 2000 [77]	Venezuela	Maternal age > 35 years (main risk factor) Multiparity (initial association) Macrosomia associated with glucose abnormalities		Population is described as mostly middle- and low-income
Corrêa 2004 [34]	Brazil	–	–	Late initiation of prenatal care
Forsbach 1997 [31]	Mexico	Obesity; family history of diabetes	–	–
Belmar 2004 [33]	Chile	Maternal age > 30 years	–	–
Nava 2011 [84]	Mexico	Pregestational overweight Pregestational obesity Maternal age Multiparity Family history of diabetes		Public hospital population with free medical care
Buteler-Maria 2013 [35]	Argentina	Overweight and obesity, Family history of diabetes first-degree, Macrosomia	–	–
Rodríguez Fernández 2014 [40]	Cuba	Higher maternal age, multiparity	–	–
Guimaraes 2014 [38]	Brazil	Reduced flow-mediated dilation, endothelial dysfunction	–	–
Flores-Padilla 2014 [37]	Mexico	Overweight and obesity Increased BMI Multiparity ≥25 years old Family history of type 2 diabetes Previous baby with macrosomia	–	–
Monroy 2014 [39]	Mexico	Thr130Ile polymorphism of the HNF4A gene Higher maternal age Higher BMI Elevated fasting glucose	–	–
deMelo 2015 [42]	Brazil	Higher maternal age Higher BMI Hypertension Family history of diabetes	–	–
Alves 2013 [36]	Brazil	Pre-pregnancy BMI; multiparity	–	Low educational attainment; low income
Cruz 2015 [41]	Cuba	Initial overweight or obesity; excess gestational weight gain; inadequate glycemic control; hypertriglyceridemia Elevated fetal abdominal circumference		
Reginatto 2016 [46]	Brazil	Higher Placental mass	–	–
Barbieiri 2016 [44]	Brazil	Pre-pregnancy BMI higher than 26 kg/m^2^; prior GDM; family history of diabetes	high dietary fat composition; high fatty acid intake patterns; less Physical activity; smoking	schooling less than 12 years; low socioeconomic class
Nascimento 2016 [45]	Brazil	Higher maternal age; overweight and obesity; family history of diabetes		Low income; low educational attainment
Gascho 2017 [47]	Brazil	Obesity; primigravity; previous Cesarean section	–	–
Larrabure-Torrealva 2015 [43]	Peru	Mid-pregnancy obesity; family history of diabetes; advanced maternal age	Depression during pregnancy	employment status; education level
Ruiz-Hoyos 2018 [50]	Colombia	High BMI	–	–
Aldrighi 2018 [48]	Brazil	Maternal Age advanced older than 35 years old; GDM in previous pregnancy	No prenatal visits, a few prenatal visits	low educational attainment, married or stable union, employed in low-income occupations, and resided outside the municipality.
Guerra 2018 [49]	Brazil	Pre-gestational overweight and pre-gestational obesity	–	–
Camarena-Pulido 2018 [78]	Mexico	Low vitamin D (<30 ng/mL) associated with GDM Higher BMI Older maternal age	–	–
Dantas 2019 [54]	Brazil	higher parity, higher BMI	–	–
Tuesca Molina 2019 [61]	Colombia	Age > 25 years Obesity Previous macrosomia Previous GDM First-degree family history of diabetes Abnormal glucose tolerance history Polycystic ovary syndrome Hypertension Steroid use Abnormal birth weight (maternal at birth) Excessive weight gain in 2nd/3rd trimester	–	–
Villota-Burbano 2019 [62]	Mexico	Age (older), Overweight/obesity, Family history of diabetes (first degree), Previous GDM	–	–
Barros 2019 [51]	Brazil	Maternal age ≥ 25 years	–	–
Siqueira 2019 [60]	Brazil	Family history of GDM high BMI Older Age	–	–
Freitas 2019 [55]	Brazil	Maternal age ≥ 35 years, Pregestational overweight, Obesity, Family history of type 2 diabetes	–	–
Martinez-Cruz 2019 [57]	Mexico	Pre-gestational obesity, Higher fasting glucose, Family history of diabetes	–	–
Correa 2019 [53]	Chile	Elevated in first trimester: Total cholesterol LDL cholesterol Triglycerides Insulin HOMA-IR t-PA Tissue plasminogen activator	–	–
Hernández 2019 [56]	Honduras	Family history of diabetes mellitus	–	–
Pent 2019 [59]	Argentina	Obesity, vitamin D deficiency	–	–
Preciado 2020 [64]	Colombia	Elevated BMI	–	–
DosSantos 2020 [63]	Brazil	Maternal age ≥ 35 years Overweight (BMI ≥ 25 kg/m^2^) Higher parity (≥3 pregnancies)	–	–
Nicolosi 2020 [79]	Brazil	Maternal age ≥ 25 years BMI ≥ 25 kg/m^2^ Family history of diabetes Multiparity Hypertension Elevated HbA1c	Sedentarism/nonexercised Weight gain during pregnancy	Employment status Partner status
Collantes-Gutiérrez 2020 [80]	Mexico	Vitamin D deficiency BMI Maternal age Family history of diabetes		
Pagotto 2020 [81]	Argentina	Obesity Maternal age		Discussion of public vs private healthcare and socioeconomic differences
Argerich 2021 [67]	Argentina	First-trimester fasting glycemia > 85 mg/dL Pregestational BMI < 24.9 BMI > 30 Advanced Maternal age High Maternal triglyceride levels	–	–
Olivares 2021 [68]	Argentina	Overweight and obesity; family history of diabetes; elevated fasting glucose at diagnosis; elevated blood pressure; previous GDM; fetal macrosomia	–	Health system barriers
Almeida 2021 [66]	Brazil	Pregestational BMI overweight, obesity; hypertension, Parity; personal history of GDM; family history of diabetes	Low Physical Activity, low DQI-R scores in whole grains, oils, milk dairy products, soy beverages, high consumption of sodium and saturated fats	Low educational level
Pagotto 2022 [69]	Argentina	Advanced maternal age; obesity; progressive insulin resistance with advancing gestation		
Carvalho 2022 [82]	Brazil	Neck circumference Older maternal age Overweight/obesity Blood pressure Hyperglycemia	Physical activity	Education
Juvinao-Quintero 2023 [70]	Peru	Overweight and obesity; family history of diabetes	–	Food insecurity Educational level low unemployment status
Zavalza-Gomez 2023 [73]	Mexico	Higher pregestational BMI, Multiparity (≥3 pregnancies), Family history of diabetes, Higher birth weight ≥ 3000 g	–	–
Rovira 2023 [72]	Argentina	BMI > 25 kg/m^2^; family history of diabetes; history of macrosomia	–	–
Zulueta 2023 [83]	Mexico	Genetic polymorphisms (11 SNPs) Maternal age Pre-pregnancy BMI Family history of T2D Previous pregnancies	Diet Sleep duration Physical activity interactions with MTNR1B variants	
Garcia-Alvarado 2024 [74]	Mexico	Advanced maternal age; higher pregestational weight and BMI; family history of type 2 diabetes, multiparity; higher levels of: Triglycerides Oxidative stress marker (MDA) Antioxidant enzymes (CAT, SOD)	Higher intake of simple carbohydrates (sucrose)	Rural residence Low to middle educational level
Gurza 2025 [75]	Mexico	Elevated TyG index (>8.6); higher pregestational BMI; higher pregestational weight; higher fasting glucose and triglycerides; insulin resistance	–	–
Perichart-Perera 2025 [76]	Mexico	Pregestational BMI; higher HOMA-IR; higher Fasting glucose; higher Insulin levels; higher Free fatty acids (FFA); higher Gestational weight gain (GWG)		

Other recurrent biological determinants of GDM risk included multiparity, previous GDM, prior macrosomia, hypertension, and metabolic abnormalities such as elevated fasting glucose, insulin resistance, dyslipidemia, and altered inflammatory or oxidative markers [29,39,42,53,69,74,75].

Behavioral determinants were less frequently documented but encompassed unhealthy dietary patterns characterized by high intake of fats or simple carbohydrates, also low physical activity, smoking, depression during pregnancy, and limited adherence to nutritional counseling or therapeutic regimens [43,44,66,74]. Some studies also highlighted inadequate prenatal care utilization, including delayed or limited prenatal visits [34,48].

Social determinants were also identified in several studies. These included low educational attainment, low socioeconomic status, food insecurity, unemployment, and barriers related to health system access or postpartum follow-up [36,44,45,48,66,68,70,74]. Rural residence and limited awareness of GDM prevention and self-care practices were also reported [74].

## Discussion

### Main findings

This scoping review identified 56 observational studies examining the epidemiology and determinants of GDM in Latin America and the Caribbean. Reported prevalence estimates ranged widely, from 3.0% to 61.0%, while incidence estimates varied from 3.2% to 47.9%, highlighting substantial heterogeneity across studies. Advanced maternal age, elevated BMI, family history of diabetes, previous GDM, prior macrosomia, and hypertension emerged as the most consistently reported risk factors, whereas behavioral and social determinants were assessed less frequently.

The substantial variability observed in prevalence and incidence estimates is likely driven by multiple interacting factors, including differences in study populations, healthcare settings, diagnostic criteria, screening strategies, and underlying populations risk profiles. Studies conducted in tertiary referral centers and high-risk obstetric clinics generally reported higher prevalence estimates than population-based investigations, reflecting differences in baseline risk. Notably, the highest prevalence estimates generally were reported in relatively small studies and selected high risk population. These differences were further amplified by substantial heterogeneity in diagnostic criteria, screening strategies, and definitions of key risk factors across studies.

IADPSG and WHO-2013 recommendations are largely comparable because WHO-2013 adopted the same 75-g OGTT glucose thresholds proposed by IADPSG (fasting ≥ 92 mg/dL, 1-hour ≥ 180 mg/dL, and 2-hour ≥ 153 mg/dL) [26,27]. In contrast, ADA recommendations allowed both a one-step approach based on IADPSG thresholds and a two-step approach involving a 50-g glucose challenge test followed by a diagnostic 100-g OGTT using Carpenter-Coustan criteria [85]. Furthermore, some studies implemented universal screening whereas others relied on risk-based screening strategies, potentially contributing to differences in case ascertainment and prevalence estimates [28]. The epidemiological impact of these methodological differences was illustrated by a Chilean study included in this review, in which GDM incidence increased from 3.2% using WHO criteria to 7.7% using ADA criteria [33]. Additional heterogeneity arose from inconsistent definitions of key risk factors. Advanced maternal age was defined using thresholds ranging from ≥25 years to ≥35 years, whereas several studies analyzed age as a continuous variable or reported only mean age values [37,55,61]. Similarly, BMI was assessed using pre-pregnancy BMI, early-pregnancy BMI, or BMI measured during pregnancy and was reported either categorically (overweight ≥ 25 kg/m^2^ and obesity ≥ 30 kg/m^2^) or continuously. Such variability limits comparability across studies and complicates interpretation of the epidemiological evidence across the region.

Across studies, advanced maternal age was consistently identified as a risk factor for GDM [37,55,61]; however, the thresholds used to define this exposure varied substantially, ranging from ≥25 years in some studies [37,61] to >30 years [33] and >35 years in others [48,55,63,77], while several studies referred only to ‘advanced’ or ‘higher’ maternal age without specifying a cutoff [29,32,39,40,42,45,62,78,80,84]. Similar heterogeneity was observed in adiposity measures. BMI was assessed as pre-pregnancy BMI [36,44,49,55,57,84], early-pregnancy or initial BMI [41], mid-pregnancy obesity [43], or simply reported as elevated BMI without specification of timing [37,39,42,50,54,60,64,78,80]. Moreover, some studies used categorical definitions of being overweight or obesity, whereas others analyzed BMI as a continuous variable or used alternative thresholds such as BMI > 26 kg/m^2^ [44]. Such variability limits comparability across studies and constrains the ability to generate pooled inferences.

### Comparison with other studies

Our findings are consistent with the wider epidemiological literature, which highlights ongoing challenges in accurately diagnosing and screening for GDM globally [86–88]. For many years, the lack of consensus on diagnostic criteria has impeded the accurate estimation of both the prevalence and incidence of GDM, a challenge that persists in current evidence. Furthermore, while the metabolic pathways that contribute to the development of GDM are not yet fully understood [20,89–92], its role as a significant risk factor for adverse maternal and offspring health outcomes is well established [28,93,94].

The pattern of risk factors identified in LAC such as advanced maternal age, elevated BMI, family history of diabetes, and prior GDM aligns closely with global evidence [86,95,96]. Similar to observations from other world regions, differences in diagnostic thresholds, screening strategies, ethnicity, migration patterns, and underlying populations risk profiles contribute substantially to variability in reported prevalence estimates [97–104].

Studies among migrant and ethnically diverse populations further suggest that GDM risk is shaped by a complex interaction between genetic susceptibility, obesity prevalence, healthcare access and sociocultural factors [101–103]. However, evidence evaluating these determinants within LAC populations remains limited.

### Evidence gaps

This review also highlights several important evidence gaps. Despite the growing burden of metabolic disease in the region, most available evidence remains observational and originates from a limited number of countries, particularly Brazil and Mexico, while Caribbean nations and several Central American Countries remain markedly underrepresented [8,105]. Prospective population-based cohorts were uncommon, limiting assessment of temporal relationships and long-term maternal and offspring outcomes.

Considerable heterogeneity in diagnostic criteria, exposure definitions, and outcome reporting further complicates comparisons across studies. In particular, few investigations assessed social determinants such as education, income, food insecurity, access to prenatal care, or rural–urban disparities despite their potential relevance in highly unequal settings such as LAC [106]. Future research would benefit from harmonized diagnostic approaches, standardized measurement of maternal age and BMI, and more comprehensive evaluation of behavioral and social determinants as well as postpartum outcomes [107–111].

### Research, policy, and clinical implications

While additional epidemiological evidence and greater diagnostic harmonization remain important regional priorities, several actions can already be implemented across healthcare systems in LAC.

Evidence gap, particularly in underrepresented Caribbean and Central American Countries, highlight the need for more prospective population-based studies and more standardize research methods to improve the comparability and generalizability of findings.

At the policy level, the substantial heterogeneity in prevalence estimates, screening strategies, and diagnostic criteria underscore the need for greater regional harmonization. Coordinated efforts through ALAD, PAHO, and national ministries of health could improve surveillance and comparability across countries, and support evidence-informed maternal health policies and resource allocation.

From a clinical perspective, maternal overweight or obesity, advanced maternal age, previous GDM, family history of diabetes, and previous macrosomia emerged were the most consistently reported risk factors across studies included in this review. Early prenatal visits may offer an opportunity to identify women at increased risk and prioritize preventive interventions [10,86,112]. Primary care providers and maternal health services can promote healthy dietary patterns, physical activity, and appropriate gestational weight gain while increasing attention to clinical indicators of dysglycemia during pregnancy, particularly among women with cardiometabolic risk factors [113,114]. Strengthening postpartum glucose testing and continuity of care after delivery may reduce progression to type 2 diabetes and help interrupt the intergenerational transmission of cardiometabolic risk [86,113].

### Strengths and limitations of the study

This is to our knowledge, the first scoping review to comprehensively map the available observational evidence on the prevalence, incidence, and determinants of GDM in LAC, as well as the methodological characteristics of the included observational studies. The included studies covered multiple countries, healthcare settings, and study designs, allowing for a broad characterization of GDM epidemiology across the region. By systematically synthesizing biological, behavioral, and social determinants, this review contributes to understanding the multifactorial nature of GDM and identifies important priorities for future research and public health action.

Nevertheless, several limitations should be acknowledged. First, substantial heterogeneity in diagnostic criteria, screening strategies, and exposure definitions limited comparability across studies. Second, inconsistent reporting of prevalence, incidence, and risk factors resulted in incomplete evidence across some domains. Third, the predominance of observational and frequently hospital-based studies limits causal inference and may overestimate disease burden relative to the general population. Fourth, variation in measurement methods and reporting practices across countries may have affected the consistency of the available literature rather than reflecting shortcomings in the review methodology itself. Finally, evidence from Caribbean and several Central American countries was sparse. Consequently, the findings may not fully reflect the epidemiology of GDM across all countries in the region and should be interpreted considering these geographical evidence gaps.

## Conclusion

Available evidence suggests that GDM is a significant maternal health concern across LAC countries, however, substantial heterogeneity in prevalence estimates, ranging from 3% to 61%, limits precise assessment of its regional burden. This variability reflects differences in diagnostic criteria, screening strategies, study populations, and healthcare settings. Nevertheless, the consistent identification of GDM across countries and clinical setting underscore its relevance as regional public health issue.

Early prenatal visits provide an opportunity to identify women at increased risk based on maternal age, overweight or obesity, previous GDM, family history of diabetes, hypertension, or previous macrosomia [10,86]. While the present review did not evaluate the effectiveness of preventive interventions, the identified risk factors profile has implications for prevention and management. Based on the broader evidence and according to the current clinical recommendations, primary care providers and maternal health services can promote healthy dietary patterns and physical activity and monitor gestational weight gain from the beginning of pregnancy, particularly among women with cardiometabolic risk factors [113,114]. Improving postpartum glucose testing and continuity of care after delivery may reduce progression to type 2 diabetes.

Important evidence gaps remain. The current literature is based on observational studies, particularly cross-sectional designs, and substantial heterogeneity in screening and diagnostic approaches limit direct comparison across studies. Future research should prioritize prospective population-based studies, harmonization of diagnostic criteria, and evaluation of context-specific prevention and management strategies to support evidence-informed policy and practice in the region.

## Supplementary Material

PRISMA checklist.docx

Supplementary_material_1.docx

## Data Availability

No new data was generated or analyzed in support of this research. Data sharing is not applicable to this article as no datasets were generated or analyzed during the current stage. The study protocol has been preregistered on the Open Science Framework (OSF) and is publicly accessible at [(DOI: 10.17605/OSF.IO/KNJZC]

## Abbreviations

ADA: American Diabetes Association
ALAD: Latin American Diabetes Association
BMI: Body Mass Index
DeCS: Descriptores en Ciencias de la Salud in Spanish
Emtree: Excerpta Medica Tree
FFA: Free Fatty Acids
GDM: Gestational Diabetes Mellitus
GWG: Gestational Weight Gain
HNF4A: Hepatocyte Nuclear Factor 4 Alpha
HOMA-IR: Homeostatic Model Assessment of Insulin Resistance
IADPSG: International Association of Diabetes and Pregnancy Study Groups
IMSS: Instituto Mexicano del Seguro Social
INMP: Instituto Nacional Materno Perinatal
INPer: Instituto Nacional de Perinatología
JBI: Joanna Briggs Institute
LAC: Latin America and the Caribbean
LDL: Low-Density Lipoprotein
LILACS: Latin American and Caribbean Health Sciences Literature
MeSH: Medical Subject Headings
NR: Not Reported
OGTT: Oral Glucose Tolerance Test
PCOS: Polycystic Ovary Syndrome
PRISMA-ScR: Preferred Reporting Items for Systematic Reviews and Meta-Analyses Extension for Scoping Reviews
SciELO: Scientific Electronic Library Online
SEDENA: Secretaría de la Defensa Nacional
SUS: Sistema Único de Saúde
T2D: Type 2 Diabetes
TyG: Triglyceride-Glucose Index
UFMG: Universidade Federal de Minas Gerais
UFRJ: Federal University of Rio de Janeiro
UNIFESP: Federal University of São Paulo
WHO: World Health Organization

## Open scholarship

This article has earned the Center for Open Science badge for Preregistered. The materials are openly accessible at https://doi.org/10.17605/OSF.IO/KNJZC
