# Supplementary material for: Epidemiology of gestational diabetes mellitus in Latin America and the Caribbean: a scoping review
Source: Glob Health Action. 2026 Nov 10;19(1):2759388. doi: 10.1080/16549716.2026.2759388 (PMC13757914; doi:10.1080/16549716.2026.2759388)
Supplement: Supplementary file 1 [file ZGHA_A_2759388_SM0494.docx]

**Supplementary material 1:** Full Search Strategy

| Data Base | Searches |
| --- | --- |
| Embase | (exp gestational diabetes/ OR ((diabetes OR mellitus) adj3 (pregnanc* OR pregnant OR gestational)).mp. OR GDM.mp.) AND (exp "South and Central America"/ OR latin america.mp. OR Mexico.mp. OR ("Antigua and Barbuda" OR Argentina OR Bahamas OR Barbados OR Belize OR Bolivia OR Brazil OR Chile OR Colombia OR "Costa Rica" OR Cuba OR Dominica OR "Dominican Republic" OR Ecuador OR "El Salvador" OR Grenada OR Guatemala OR Guyana OR Haiti OR Honduras OR Jamaica OR Nicaragua OR Panama OR Paraguay OR Peru OR "Saint Kitts and Nevis" OR "Saint Lucia" OR "Saint Vincent and the Grenadines" OR Suriname OR "Trinidad and Tobago" OR Uruguay OR Venezuela).mp.) |
| MEDLINE (via Ovid) | (exp Diabetes, Gestational/ OR ((diabetes OR mellitus) adj3 (pregnanc* OR pregnant OR gestational)).mp. OR GDM.mp.) AND (exp central america/ OR exp south america/ OR latin america.mp. OR central america.mp. OR south america.mp. OR Mexico.mp. OR ("Antigua and Barbuda" OR Argentina OR Bahamas OR Barbados OR Belize OR Bolivia OR Brazil OR Chile OR Colombia OR "Costa Rica" OR Cuba OR Dominica OR "Dominican Republic" OR Ecuador OR "El Salvador" OR Grenada OR Guatemala OR Guyana OR Haiti OR Honduras OR Jamaica OR Nicaragua OR Panama OR Paraguay OR Peru OR "Saint Kitts and Nevis" OR "Saint Lucia" OR "Saint Vincent and the Grenadines" OR Suriname OR "Trinidad and Tobago" OR Uruguay OR Venezuela).mp.) |
| Web of Science (WoS | TS=(("gestational diabetes" OR GDM OR (pregnanc* NEAR/3 diabetes)) AND ("Latin America" OR "Central America" OR "South America" OR Mexico OR Argentina OR Bolivia OR Brazil OR Chile OR Colombia OR "Costa Rica" OR Cuba OR Dominica OR "Dominican Republic" OR Ecuador OR "El Salvador" OR Grenada OR Guatemala OR Guyana OR Haiti OR Honduras OR Jamaica OR Nicaragua OR Panama OR Paraguay OR Peru OR "Saint Kitts and Nevis" OR "Saint Lucia" OR "Saint Vincent and the Grenadines" OR Suriname OR "Trinidad and Tobago" OR Uruguay OR Venezuela OR "Antigua and Barbuda" OR Bahamas OR Barbados OR Belize)) |
| SciELO | ((Diabetes gestacional) OR (Diabetes Mellitus Gestacional) OR (Diabetes Inducida por Embarazo) OR (Diabetes Induzida pela Gravidez) OR (Diabetes Induzida por Gravidez)) AND ((América Latina) OR (Latinoamérica) OR (Antigua y Barbuda) OR (Argentina) OR (Bahamas) OR (Barbados) OR (Belice) OR (Bolivia) OR (Brasil) OR (Chile) OR (Colombia) OR (Costa Rica) OR (Cuba) OR (Dominica) OR (Ecuador) OR (El Salvador) OR (Granada) OR (Guatemala) OR (Guyana) OR (Haití) OR (Honduras) OR (Jamaica) OR (México) OR (Nicaragua) OR (Panamá) OR (Paraguay) OR (Perú) OR (República Dominicana) OR (San Cristóbal y Nieves) OR (San Vicente y las Granadinas) OR (Santa Lucía) OR (Suriname) OR (Trinidad y Tobago) OR (Uruguay) OR (Venezuela)) |
| LILACS | ((Diabetes gestacional) OR (Diabetes Mellitus Gestacional) OR (Diabetes Inducida por Embarazo) OR (Diabetes Induzida pela Gravidez) OR (Diabetes Induzida por Gravidez)) AND ((América Latina) OR (Latinoamérica) OR (Antigua y Barbuda) OR (Argentina) OR (Bahamas) OR (Barbados) OR (Belice) OR (Bolivia) OR (Brasil) OR (Chile) OR (Colombia) OR (Costa Rica) OR (Cuba) OR (Dominica) OR (Ecuador) OR (El Salvador) OR (Granada) OR (Guatemala) OR (Guyana) OR (Haití) OR (Honduras) OR (Jamaica) OR (México) OR (Nicaragua) OR (Panamá) OR (Paraguay) OR (Perú) OR (República Dominicana) OR (San Cristóbal y Nieves) OR (San Vicente y las Granadinas) OR (Santa Lucía) OR (Suriname) OR (Trinidad y Tobago) OR (Uruguay) OR (Venezuela)) |
